# Targeted inhibition of Focal Adhesion Kinase Attenuates Cardiac Fibrosis and Preserves Heart Function in Adverse Cardiac Remodeling

**DOI:** 10.1038/srep43146

**Published:** 2017-02-22

**Authors:** Jie Zhang, Guangpu Fan, Hui Zhao, Zhiwei Wang, Fei Li, Peide Zhang, Jing Zhang, Xu Wang, Wei Wang

**Affiliations:** 1 Department of Cardiovascular Surgery, State Key Laboratory of Cardiovascular Disease, Fuwai Hospital, National Center for Cardiovascular Diseases, Chinese Academy of Medical Sciences and Peking Union Medical College, Beijing, China; 2 Department of Cardiovascular Surgery, Peking University People’s Hospital, Beijing, China

## Abstract

Cardiac fibrosis in post-myocardial infarction (MI), seen in both infarcted and non-infarcted myocardium, is beneficial to the recovery of heart function. But progressively pathological fibrosis impairs ventricular function and leads to poor prognosis. FAK has recently received attention as a potential mediator of fibrosis, our previous study reported that pharmacological inhibition of FAK can attenuate cardiac fibrosis in post MI models. However, the long-term effects on cardiac function and adverse cardiac remodelling were not clearly investigated. In this study, we tried to determine the preliminary mechanisms in regulating CF transformation to myofibroblasts and ECM synthesis relevant to the development of adverse cardiac remolding *in vivo* and *in vitro*. Our study provides even more evidence that FAK is directly related to the activation of CF in hypoxia condition in a dose-dependent and time-dependent manner. Pharmacological inhibition of FAK significantly reduces myofibroblast differentiation; our *in vivo* data demonstrated that a FAK inhibitor significantly decreases fibrotic score, and preserves partial left ventricular function. Both PI3K/AKT signalling and ERK1/2 are necessary for hypoxia-induced CF differentiation and ECM synthesis; this process also involves lysyl oxidase (LOX). These findings suggest that pharmacological inhibition of FAK may become an effective therapeutic strategy against adverse fibrosis.

Myocardial fibrosis is a significant global health problem associated with disrupted tissue function and end-stage heart failure^1^. Despite the clinical importance of fibrosis in cardiovascular disease, the cell biological processes underlying fibrosis development remain relatively mischaracterized and poorly understood, emphasizing a need for potential strategies that effectively prevent its pathological contribution to adverse cardiac remodelling.

Cardiac fibroblasts (CFs) are the major constituent cells of the heart, and are the governing source of components of the extracellular matrix (ECM), which regulates the structure of the heart^2^,^3^,^4^,^5^. Fibroblast migration into the infarcted and non-infarcted myocardium has been described as one of the initial steps affecting the outcome of cardiac remodelling. Under normal conditions, there is a balance between the synthesis and degradation of extracellular matrix^6^. However, in response to myocardial injury, persistent fibroblast activation and cell phenotypic conversion from fibroblasts to myofibroblasts result in the excessive production and accumulation of ECM proteins^7^. This pathological remodelling increases ventricular stiffness, leads to arrhythmias, and ultimately affects heart function (HF)^8^,^9^,^10^,^11^.

Focal adhesion kinase (FAK) is a 125-kDa non-receptor tyrosine kinase^12^,^13^,^14^. In addition to its role in cell-to-extracellular matrix connections, it also plays a critical role in regulating cell proliferation, migration, adhesion, and survival in a wide range of cell types^15^,^16^,^17^,^18^. FAK has recently received attention as a potential mediator of fibrosis, including lung fibrosis^19^,^20^,^21^, liver fibrosis^22^, kidney fibrosis^23^, skin fibrosis^24^, muscle fibrosis^25^,^26^, and atherosclerosis^27^. Our previous study reported that FAK is involved in atrial fibrosis and that pharmacological inhibition of FAK can suppress α-SMA expression in TGFβ1-induced fibroblasts^28^,^29^ and attenuate cardiac fibrosis in post-myocardial infarction models. However, the long-term effects on cardiac function and adverse cardiac remodelling were not clearly investigated. Therefore, a comprehensive investigation is still needed on such an important active molecule in cardiac remodelling.

In this study, we performed *in vivo* research with a post-myocardial infarction (MI)-induced cardiac fibrosis model and *in vitro* investigations with CFs and tried to determine the preliminary mechanisms regulating CF transformation to myofibroblasts and ECM synthesis relevant to the development of adverse cardiac remodelling. Our study provides even more evidence that FAK is directly related to the activation of fibroblasts and phenotype conversion in hypoxia culture conditions. Pharmacological inhibition of FAK significantly decreased ECM synthesis and myofibroblast differentiation *in vitro*. Our *in vivo* data demonstrated that a FAK inhibitor significantly reduces FAK activation, decreases fibrotic score, and preserves partial left ventricular function. Both PI3K/AKT signalling and ERK1/2 are necessary for hypoxia-induced CF differentiation and ECM synthesis; this process also involves lysyl oxidase (LOX). These findings suggest that pharmacological inhibition of FAK may become an effective therapeutic strategy against adverse fibrosis.

## Results

### FAK is activated and directly associated with hypoxia-induced fibroblast activation and cell phenotypic conversion; Inhibition of FAK activation decreases hypoxia–induced fibroblast phenotypic conversion

**Figure 1:**
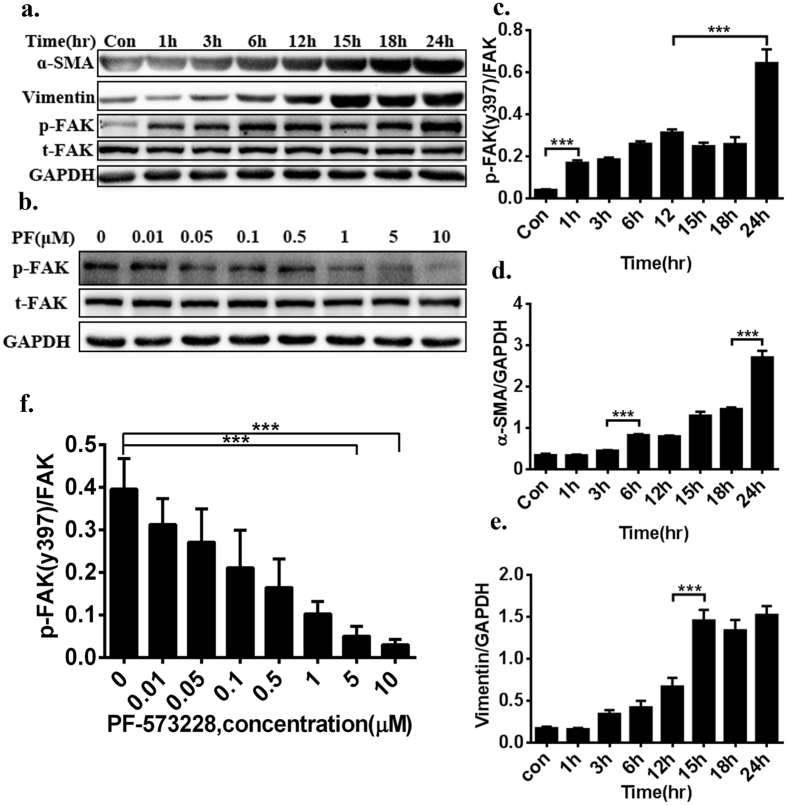
FAK is activated and directly associated with hypoxia-induced CF activation and phenotypic conversion in a dose-dependent and time-dependent manner. (**a**) α-SMA and vimentin was gradually increased with time, indicative of transformation to a myofibroblast phenotype. serum-starved and hypoxia-induced CFs had a significantly higher baseline level of p-FAK in a time-dependent manner. (**b**) Using PF-573228 at a concentration range of 0.01–10 μM for 24 hours decreased phosphorylated FAK expression in a time-dependent manner, and there was a 70% inhibition rate at the range of 5–10 μM. (**c**–**e**) Densitometric analysis of blots for determining α-SMA and Vimentin normalized to GAPDH, pY397 of FAK levels were normalized to total FAK levels. (**f**) Densitometry of inhibition level of p-FAK activation from Panel b. Multiple exposures of vimentin and GAPDH are presented in Supplementary Figure S6; full-length blots are presented in Supplementary Figure S1 (1a,1b). Data are presented as means ± SEM. ***P < 0.001.

CFs were isolated from the hearts of two- to four-day-old neonatal CD1 mice and cultured in hypoxia and serum-free conditions^30^. The α-smooth muscle actin (**α**-SMA) expression level gradually increased with time, indicative of transformation to a myofibroblast phenotype (Fig. 1a and d). As a fibroblast marker protein that is considered to be involved in phenotypic conversion, vimentin also displayed a parallel (8.7-fold) increase after treatment for 24 hours (Fig. 1a and e). We then examined the level of phosphorylated FAK tyrosine 397 (pY397 of FAK), a well-known marker of FAK activation^31^. Compared with the normal group, serum-starved and hypoxia-induced CFs had a significantly higher baseline level of p-FAK in a time-dependent manner, and the level was rapidly increased at 24 hours (Fig. 1a and c). Next, we used PF-573228 (PF), an ATP-competitive inhibitor of FAK, to inhibit the phosphorylation of FAK at a concentration range of 0.01–10 μM for 24 hours. Hypoxia-induced FAK activation occurred in a dose-dependent manner (Fig. 1b), the level of phosphorylated FAK tyrosine 397 was negatively associated with the dose used, and there was a 70% inhibition rate at the range of 5–10 μM (Fig. 1f).

**Figure 2:**
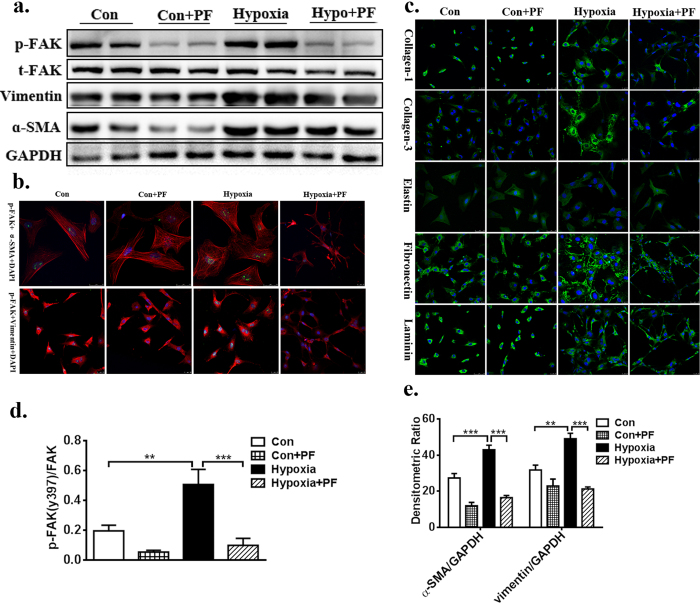
Inhibition of FAK activation decreases Hypoxia–induced CFs phenotypic conversion and ECM synthesis. (**a**) Using PF-573228 at the dose of 5 μM significantly decreased p-FAK expression level by nearly 80%, as well as vimentin expression by 47% and α-SMA expression by 60% compare to the untreated failing CFs. (**b**) Immunofluroescence show that after treatment with PF-573228, p-FAK(green), α-SMA(red) and vimentin(red) were reduced, which was indicated by lower fluorescence intensity. (**c**) Representative pictures of ECM synthesis including collagen-1, collagen-3, laminin, elastin, fibronectin with green AlexaFluor 647 dye. (**d**,**e**) Densitometric analysis of blots from panel a. Nuclei was stained blue with DAPI, magnification × 400. Full-length blots are presented in Supplementary Figure S2. Data are presented as means ± SEM. **P < 0.01 and ***P < 0.001.

To investigate the potential role of FAK in CF phenotypic conversion, CFs were cultured in a hypoxic GENbox jar fitted with a catalyst (BioMérieux) to scavenge free oxygen for 24 hours and were treated with or without the FAK inhibitor PF-573228 (5 μM). Two control groups were applied: one being an untreated control group, and the other a group containing cells incubated with PF. Using PF-573228 at the dose of 5 μM significantly decreased phosphorylated FAK expression level by nearly 80% (p < 0.001, Fig. 2a and d). Meanwhile, pharmacological inhibition of FAK decreased vimentin expression by 47% and α-SMA expression by 60% in the failing CFs (p < 0.001, Fig. 2a and e, respectively). Consistent with these findings, confocal microscopy showed that serum starvation and hypoxia can significantly change the morphology of CFs over-expressing p-FAK. After treatment with PF, α-SMA and vimentin were reduced, as indicated by the lower fluorescence intensity (Fig. 2b); pharmacological inhibition of FAK was beneficial in retaining the morphology of failing CFs. These data suggest that FAK may play an important role in the transformation of CFs into myofibroblasts *in vitro*.

### Inhibition of FAK decreases ECM synthesis in hypoxia–induced fibroblasts

**Figure 3:**
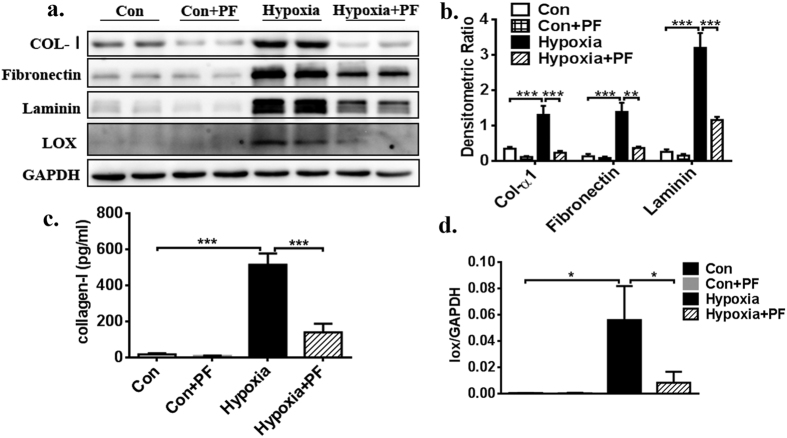
Inhibition of FAK activation decreases ECM synthesis. (**a**) Western blot evaluated expression of ECM including collagen-1, laminin, fibronectin, as well as LOX. (**b**) Densitometric analysis of blots for determining ECM normalized to GAPDH. (**c**) Cell culture medium supernatant was collected and analyzed using a Collagen I alpha 1 ELISA Kit, FAK inhibitor significantly decreased total collagen level in the hypoxia-cultured CFs compared with hypoxia-cultured CFs treated with vehicle only(514.35 ± 63.61 VS. 139.11 ± 48.57 pg/ml). (**d**) Densitometric analysis of blots for determining LOX to GAPDH. Multiple exposures of LOX are presented in Supplementary Figure S6; full-length blots are presented in Supplementary Figure S3. Data are presented as means ± SEM. *P < 0.05, **P < 0.01, ***P < 0.001.

**Figure 4:**
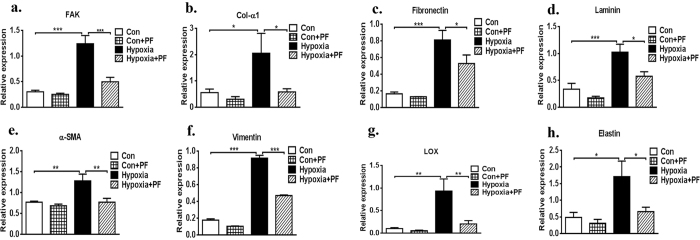
RT-PCR analysis on cardiac fibroblasts. FAK inhibitor significantly decreased total ECM production, as well as FAK, α-SMA, vimentin in hypoxia-cultured CFs relative to that in vehicle-treated CFs. Data are presented as means ± SEM. *P < 0.05,**P < 0.01,***P < 0.001.

To determine the functional role of FAK in the development of fibrosis, the effect of drug treatment on fibrosis was evaluated via ECM components, including collagen-1 (Col-1), collagen-3 (Col-3), laminin, elastin, and fibronectin. Both protein immunoblotting and confocal microscopy showed increased ECM expression in hypoxia-induced fibroblasts when compared with the control vehicle fibroblasts in the untreated state (p < 0.001, Figs 2c and 3a). Pharmacological inhibition of FAK indicated a significantly decreased fibrotic score in the hypoxia-cultured CFs relative to that in hypoxia-cultured CFs treated with vehicle only (Fig. 3a and b). There was no significant difference in ECM synthesis in the CFs treated with PF-573228 and the CFs treated with control vehicle in normal culture conditions (Fig. 3a and b). To detect the secreted collagen content, the cell culture supernatant was collected and analysed using a Collagen I alpha 1 ELISA Kit. As indicated in Fig. 3c, the FAK inhibitor significantly decreased the total collagen level in the hypoxia-cultured CFs relative to that in hypoxia-cultured CFs treated with vehicle only (514.35 ± 63.61 pg/ml VS. 139.11 ± 48.57 pg/ml, p < 0.001). RT-PCR also supported this hypothesis, as the FAK inhibitor significantly decreased the RNA expression of total ECM components, including col-1α1 (p < 0.5), laminin (p < 0.5), elastin (p < 0.5), and fibronectin (p < 0.5), as well as FAK (p < 0.001), α-SMA (p < 0.01), and vimentin (p < 0.001) in hypoxia-cultured CFs relative to that in vehicle-treated CFs (Fig. 4), demonstrating that the pharmacological inhibition of FAK significantly decreases ECM synthesis and attenuates the fibrosis level. Interestingly, no differences in baseline extracellular matrix content were observed between CFs treated with the FAK inhibitor in normal culture conditions. These above studies in normal and hypoxia-induced CFs support the hypothesis that FAK plays an important role in regulating extracellular matrix synthesis in the setting of hypoxia-induced CF phenotypic conversion.

### Inhibition of FAK restores downstream signalling in failing cardiac fibroblasts

**Figure 5:**
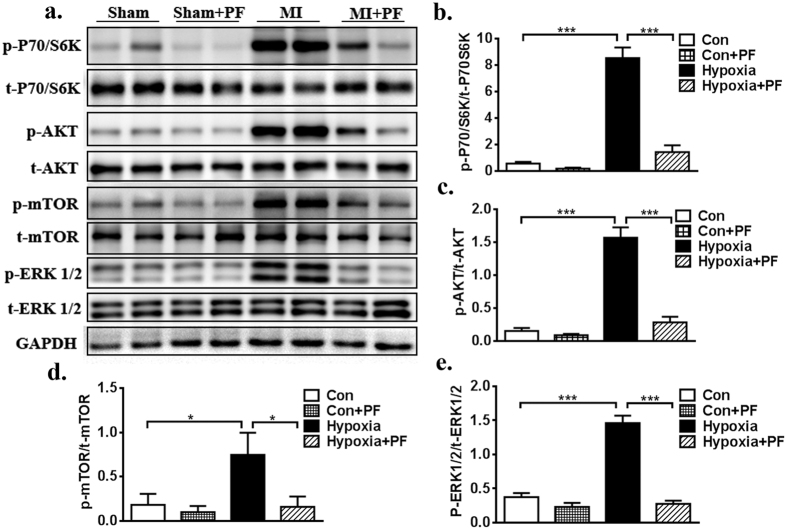
Inhibition of FAK restores downstream signaling. (**a**) Expressions of FAK-related signaling pathway including p-mTOR, p-AKT (S473), p-P70S6K, p-ERK1/2 and t-mTOR, t-AKT (S473), t-P70S6K, t-ERK1/2 in different groups (two individual samples were loaded in each group) were tested by Western Blot. (**b**–**e**) Densitometric analysis of blots from panel a. Multiple exposures of blots are presented in Supplementary Figure S6; full-length blots are presented in Supplementary Figure S5. Data are presented as means ± SEM. *P < 0.05, **P < 0.01, ***P < 0.001.

Previous studies have demonstrated that FAK downstream signalling mainly involves the phosphatidylinositol 3-kinase/protein kinase B (PI3K/AKT) signalling pathway and the extracellular signal-regulated kinase 1/2 (ERK1/2) signalling pathway^14^,^32^,^33^,^34^,^35^,^36^. PI3K/AKT is known to be a target of the rapamycin/S6 kinase (mTOR/S6K) complex, while ERK1/2 is an important cytosolic transcription factor; both are involved in cell proliferation, migration, and adhesion^37^,^38^,^39^. Therefore, we wanted to evaluate whether both signalling pathways might participate in FAK-mediated myofibroblast transformation and ECM synthesis in CFs. Western blot confirmed that hypoxia could significantly increase p-mTOR, p-AKT (S473), p-P70S6K, and p-ERK1/2 expression. Furthermore, inhibition of FAK by pretreatment with PF significantly decreased the expression of the above-described molecules (Fig. 5). In addition, there was no significant difference in the expression of these molecules between the CFs treated with PF and the CFs treated with control vehicle in normal culture condition (Fig. 5). Furthermore, RT-PCR analysis also showed that hypoxia increased the mRNA level of LOX in CFs (Fig. 4g, p < 0.01), but inhibiting FAK via pretreatment with PF significantly decreased the mRNA expression level of LOX; this trend was also confirmed with a Western blot (Fig. 3a, p < 0.5). Thus, these studies appear to support the hypothesis that FAK plays an important role in the regulation of cell phenotypic conversion and ECM synthesis under baseline conditions and in response to hypoxia stimulation via both the PI3K/AKT and ERK1/2 signalling pathways, which also involved LOX.

### A FAK inhibitor attenuates cardiac fibrosis and preserves heart function *in vivo*

**Figure 6:**
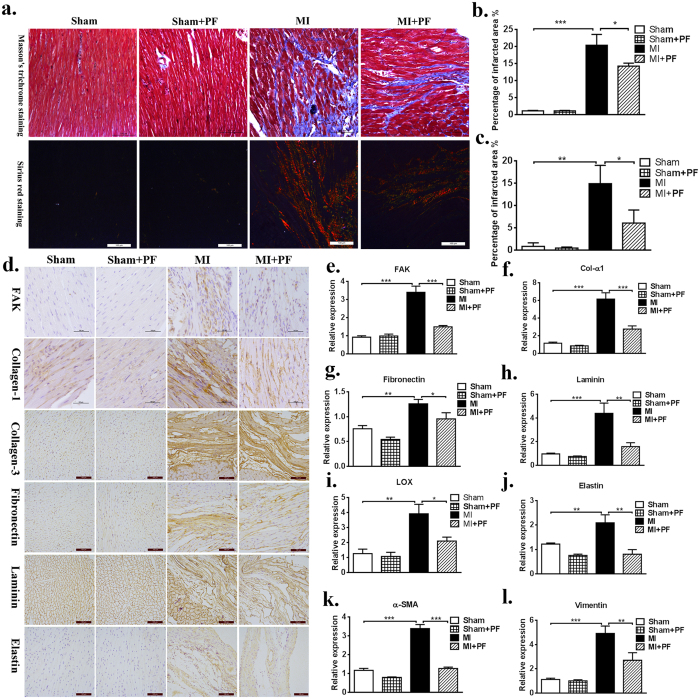
Targeted inhibition of FAK attenuates cardiac fibrosis. (**a**) Masson’s trichrome staining: in the sham group, nearly no area was stained blue. Sirius red staining: type I collagen performed red to yellow, type III collagen performed green and normal tissue performed gray. More collagens could be seen in the MI group with infiltration to non-fibrotic tissues; MI with PF reduced the area of interstitial fibrosis. (**b**,**c**) Quantitative data showing the percentage of collagen-positive area from panel a. (**d**) Immunohistochemistry: with specific primary antibody of interest, increased expression of fibrosis level can be noted at the extracellular matrix in the MI group. (**e**–**i**) RT-PCR analysis shows that FAK inhibitor significantly decreased total ECM production, as well as FAK, α-SMA, vimentin in MI with PF group when compared to that in MI group. Bar = 100 μm. Data are presented as means ± SEM. *P < 0.05, **P < 0.01, ***P < 0.001.

To further investigate the functional role of FAK in the process of post-MI interstitial fibrosis, the effect of a FAK inhibitor on post-MI fibrosis was evaluated using the MI model. Three months after surgical left anterior descending coronary artery ligation treatment, the fibrotic score determined via Masson’s trichrome staining and Sirius red staining was significantly higher in the MI group, indicating that more collagen was observed in the MI group, with infiltration to non-fibrotic tissues. However, treatment with PF protected against fibrosis in the MI animal model (Fig. 6a). There was no difference in basic ECM synthesis observed in animals treated with the FAK inhibitor or control vehicle in the sham group. Immunohistochemical analysis also showed markedly increased expression of FAK, Col-1, Col-3, fibronectin, laminin, and elastin at the extracellular matrix of the border zone in the MI group compared to the control group (Fig. 6d), suggesting that the ECM was excessively synthesized after MI. After continuous intervention with the FAK inhibitor PF-573228, ECM expression was significantly attenuated. This trend was also confirmed with RT-PCR (Fig. 6e–l). These findings suggest that the FAK inhibitor PF-573228 significantly attenuates fibrosis in the post-MI model.

To assess the effects of the FAK inhibitor on cardiac function in mice subjected to MI, the left ventricular dimensions and functional parameters were measured with echocardiography at the following time-points: 1 month, 2 months, and 3 months. During pharmacologic administration, two mice in the MI group died of heart failure in the second and third month and one of the mice in the MI treated with PF group died of sudden death during implantation of the ALZET mini-osmotic pump in the third month; none of the mice died in the unchallenged group. No differences in left ventricular anterior wall (LVAW), left ventricular internal dimension (LVID) or left ventricular posterior wall (LVPW) were noted between the two groups treated with PF and the group treated with vehicle only (Supplemental Tables S4, S5 and S6). As shown in Table 1, intervention with PF significantly preserved the left ventricular ejection fraction (LVEF), left ventricular fractional shortening (LVFS) and the systolic left ventricular volume (LV Vol; s) compared to the MI group. Plasma brain natriuretic peptide (BNP) is considered a gold-standard biomarker in determining the severity and prognosis of heart failure. In this study, the plasma BNP level was markedly increased post-MI, while PF-573228 treatment significantly counteracted this increasing trend (515.17 ± 50.75 pg/ml VS. 399.03 ± 41.37 pg/ml, p < 0.5), indicating that this inhibitor was beneficial in preserving cardiac function.

## Discussion

Although scientists have made great efforts, myocardial infarction remains a common cause of death in industrialized nations and an increasing cause of death and morbidity in many developing countries^1^. Cardiac fibrosis after post-myocardial infarction, characterized by the excess accumulation of extracellular matrix, is an important driver of disrupted tissue function and end-stage heart failure^6^. The ECM, which consists of the basic components of the heart, includes interstitial collagens, proteoglycans, glycoproteins, cytokines, growth factors, matrikines, and proteases^40^,^41^,^42^,^43^,^44^, plays a critical role in preserving 3D geometry, architecture and ventricular function. In the normal heart, there is a balance between the synthesis and degradation of ECM, which is primarily regulated by cardiac fibroblasts. In response to heart attack, CFs are activated and transformed to a myofibroblast phenotype; this regulatory mechanism and balance is disrupted. In addition to the infarcted myocardium, reactive interstitial fibrosis at the border and in remote areas also plays a pivotal role in the development of HF following MI^45^,^46^. Excessive ECM deposition reduces ventricular compliance and contributes to diastolic dysfunction; continuous fibrosis ultimately leads to systolic dysfunction, arrhythmias, and subsequent heart failure^9^,^10^,^11^.

Previous studies have demonstrated that several cytokine and growth factor signalling pathways, such as TGF-β^47^,^48^ and angiotensin^49^, are involved in CF phenotypic transformation and ECM synthesis and fibrosis. Recently, FAK has been shown to play an important role in basal myocyte function via multiple intracellular signalling pathways. Our lab has previously demonstrated that FAK is involved in atrial fibrosis, and FAK inhibition suppresses α-SMA expression in TGFβ1-induced fibroblasts^28^,^29^. Therefore, it seems reasonable that FAK can potentially be used as a predictive risk factor in adverse cardiac remodelling.

Our study demonstrates that FAK is directly related to the activation and phenotypic conversion of fibroblasts in hypoxia and serum-starved culture conditions in a dose-dependent and time-dependent manner. Pharmacological inhibition of FAK significantly reversed the phenotypic transformation in hypoxia-induced CF, as demonstrated by decreased α-SMA and vimentin expression as well as decreased basal and hypoxia-stimulated phosphorylation of FAK tyrosine 397 (pY397 of FAK) *in vitro*. The ECM consists of a collection of extracellular molecules secreted by cells that provides structural and biochemical to support the surrounding cells and includes interstitial collagens, elastin, fibronectin, and laminin; cardiac fibroblasts are responsible for maintaining the homeostasis of the extracellular matrix. In our study, we found that hypoxia can significantly increase the expression level of ECM components, such as Col-1, Col-3, fibronectin, and laminin, while inhibition of FAK can offset this trend. Interestingly, elastin was not observed using Western blot with equal amounts of total protein, but it could be detected by immunohistochemistry and was mainly distributed in the peripheral capillaries and infarcted area, indicating that this protein showed limited expression.

Lysyl oxidase (LOX) is a copper-dependent amine oxidase that plays a critical role in the biogenesis of connective tissue matrices, mainly participating in the crosslinking of extracellular matrix proteins, such as collagen and elastin, by oxidizing peptidyl lysine in these proteins to peptidyl α-aminoadipic-8-semialdehyde^50^. As ECM synthesis increases, collagen and elastin could be cross-linked by LOX to produce more mature extracellular matrix to promote fibrosis^51^,^52^. In our study, we found that hypoxia increased the expression level of LOX in CFs, while inhibition of FAK significantly decreased the expression of LOX, indicating that LOX may be involved in FAK-related ECM synthesis.

**Figure 7:**
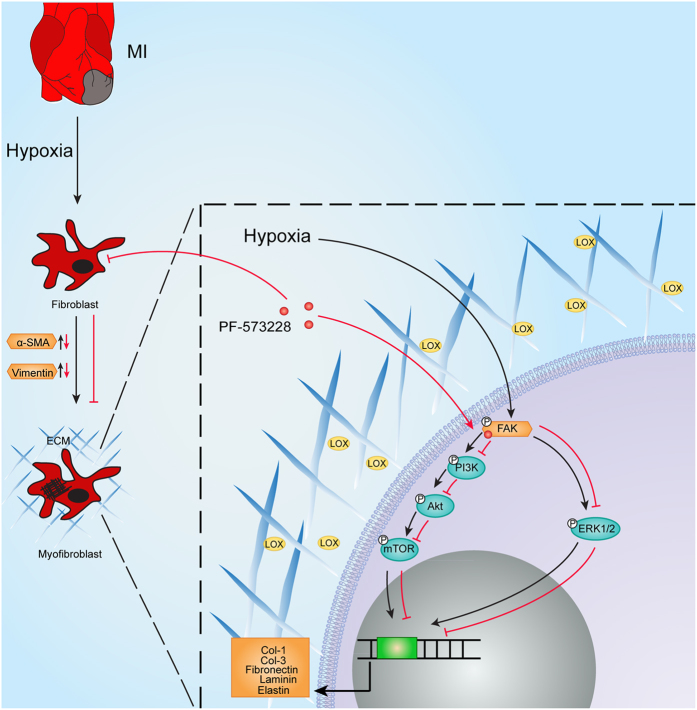
Schematic representation of FAK involvement in signaling pathways. FAK can be activated in ischemic and hypoxic conditions; its downstream PI3K/AKT pathway is then activated through phosphorylation at the Tyr397 site, followed by mTOR/P70S6K activation. ERK1/2 can also be activated simultaneously. Both PI3K/AKT signalling and ERK1/2 are necessary for hypoxia-induced CF differentiation and ECM synthesis; this process also involves lysyl oxidase (LOX). Consequently, this response could be offset by FAK inhibitor.

We previously reported that AKT/S6k was involved in CF differentiation induced by TGF-β stimulation *in vitro*, rather than the ERK1/2 signalling pathway. Our current investigation suggests that PI3K/AKT signalling and ERK1/2 signalling are both necessary for hypoxia-induced CF differentiation and ECM synthesis (Fig. 7). FAK can be activated by ischaemic and hypoxic conditions; its downstream PI3K/AKT pathway is then activated through phosphorylation at the Tyr397 site followed by mTOR/P70S6K activation, and ERK1/2 can also be activated simultaneously. These studies suggest that a more complex environment exists *in vivo*; other mechanisms could also influence the expression of relevant proteins in MI animal models. The present study might contribute to our new knowledge of cellular pathway activation.

Based on the above research, we next investigated the potential role of FAK in the regulation of cardiac fibrosis using the post-MI model. Different from the previous study, CD1 mice were used in the current study after take a full consideration of animal size, pump delivery rate, route of administration, duration of administration. Consistent with the results *in vitro*, the FAK inhibitor significantly reduced excessive reactive interstitial fibrosis at the border and in remote areas. As a common method for evaluating cardiac function, echocardiography suggests that the targeted inhibition of FAK may be beneficial in preserving partial cardiac function, such as LVEF, LVFS and systolic left ventricular volume. The plasma BNP level (an independent predictor of long-term outcome) also supports this hypothesis. In the previous study, an improved heart function was reported, but without statistic significance^29^, we also gave some explanations, such as the infarction size, and observation time, which were both adjusted in the current study. With the careful quality control in the current study, we believe the positive results in heart function improvement are reasonable. Interestingly, PF-573228 had no side effects in the sham group or on other organs throughout the course of the study, further highlighting its safety and efficacy in mediating fibrosis in response to cardiac injury.

To conclude, FAK is involved in cardiac fibrosis *in vivo* and *in vitro*, and the targeted inhibition of FAK could attenuate cardiac fibrosis and preserve partial cardiac function. Both the PI3K/AKT and ERK1/2 signalling pathways participated in this effect, which involved LOX. However, we believe that more complicated mechanisms with multiple regulators other than those found in this study may be involved in this process. In the future, genetic deletion or translational studies will greatly help to elucidate whether this inhibitory strategy is feasible in patients with cardiac injury or HF.

## Materials and Methods

The experiments designed for the present study were performed in strict accordance with the Declaration of Helsinki, and all experimental protocols were approved by The Ethics Committee of Fuwai Hospital (No. 2015-M-200-GZ). Adult mice (CD1, 6–8 weeks, male) and new-born mice (CD1, 2–4 days, male) were used in the experiments.

### Reagents and Antibodies

All cell culture reagents were purchased from Gibco/Invitrogen (Carlsbad, CA, USA). PF-573228 (a FAK inhibitor) was purchased from Selleck Chemical (Houston, Texas). Unless stated otherwise, all additional chemicals were obtained from Sigma-Aldrich (St. Louis, MO, USA). A description of the antibody is included in the Supplemental Materials (see Supplemental Table S1).

### Isolation and culture of cardiac fibroblasts

CFs were isolated from the hearts of two- to four-day-old neonatal CD1 mice and cultured as described previously^53^. Briefly, the heart was sliced and tissues were subjected to enzymatic digestion. Myocardial cells were removed by differential adhesion selection, and primary cells were purified and cultured in Dulbecco’s Modified Eagle’s Medium (DMEM) containing 10% fetal bovine serum, penicillin (100 U/ml) and streptomycin (100 μg/ml) in incubator equilibrated with 95% air/5% CO_2_ at 37 °C. Trypsin-EDTA solution (0.25%) was used for sub-culturing fibroblasts; to avoid genetic mutation and low viability, third-generation cells were used for following experiments. Cells were starved for twenty-four hours prior to treatment. CFs were treated as follows: In the first part of the experiment, the cells were cultured in a hypoxic GENbox jar fitted with a catalyst (BioMérieux) to scavenge free oxygen at a pre-established time-point. In the second part of experiment, the cells were incubated with PF at concentrations of 0.01~10 μM in hypoxic conditions. Next, the CFs were randomly divided into the following experimental groups: Group A: normal, Group B: normal + PF-573228, Group C: hypoxia, Group D: hypoxia + PF-573228.

### Establishment of MI model and treatment of different groups

MI was established by permanent left anterior descending coronary artery ligation (CAL). Briefly, mice were anesthetized with tribromoethanol and ventilated with positive pressure through the tube inserted into the trachea and attached to a small animal respirator. After intubation, left lateral thoracotomy was performed at the fourth intercostal space; the left anterior descending artery was ligated using a 7-0 polypropylene suture, while in the mice undergoing the sham operation, the ligature around the LAD was not tied. At the 7th day post-MI, echocardiography was carried out to evaluate cardiac function; a total of 20 mice with a left ventricular ejection fraction of approximately 40% were selected and randomly divided into two groups (Supplemental Table S3). The sample size for each group was the following: sham (n = 6), sham + PF (n = 6), MI (n = 10), MI + PF (n = 10). PF-573228 (20 mg/kg) was administered using the ALZET mini-osmotic pump (model 2002, DURECT Corporation, USA) on the 7th day post-MI. The mice in the control group and the MI group were given pumps filled with equal quantities of vehicle.

### Implantation of the ALZET mini-osmotic pump

PF-573228 was dissolved in 50% dimethyl sulfoxide (DMSO) and 50% polyethylene glycol 300 (PEG 300). After the mini-osmotic pumps (model 2002) were completely filled with PF or vehicle, the pump was placed in sterile saline at 37  °C overnight before implantation. Mice were lightly anesthetized, a small incision was made in the skin between the scapulae, and a small pocket was made by spreading the subcutaneous connective tissue apart with a haemostat; then, the pump was embedded into the pocket. The skin incision was closed with 4-0 sutures. Every two weeks, the empty pump was replaced with a new fully filled osmotic pump under the same anaesthesia conditions.

### Doppler Echocardiography

Echocardiographic measurements were carried out with a Vevo 770 high-resolution imaging system (VisualSonics, Toronto, ON, Canada) at the time-points of 1 month, 2 months, and 3 months. Cardiac function in each mouse was expressed as the mean value of three independent two-dimensional and M-mode measurements.

### Histological analysis

After sacrificing the mice, hearts were immediately excised and fixed in 4% paraformaldehyde and then were embedded in paraffin for histological study. Paraffin sections prepared from the hearts were stained with Masson’s trichrome staining (Sigma-Aldrich HT15-1KT, St. Louis, MO, USA) and Sirius red staining (Leagene, Beijing, China.); the procedures were mainly based on the manufacturer’s instructions. Each section was imaged under a microscope (Leica, DM600B). The ratio of interstitial fibrosis to myocardium was quantified using ImageJ software (U.S. National Institutes of Health, Bethesda, MD, USA).

### Immunohistochemical staining

Tissue sections were baked for 45 min at 68 °C, deparaffinized, rehydrated and subjected to antigen retrieval. Endogenous peroxidase activity was quenched with 3% H_2_O_2_ for 10 min. The sections were washed (3×) in phosphate-buffered saline (PBS, 5 min) and successively blocked with appropriate serum (30 min, room temperature). The tissue sections were incubated overnight with primary antibodies of interest (4 °C), rewarmed (37 °C) for 45 min, washed (3×) with PBS (5 min), incubated with horseradish peroxidase-conjugated secondary antibody (1 h, room temperature), and washed (3×) with PBS (5 min). As a negative control, primary antibody was omitted from the staining reactions. The DAB colour reaction was monitored, slide-by-slide, under the microscope to avoid error from background light.

### Confocal microscopy

CFs were grown on plates, fixed with 4% PFA for 15 min, permeabilized with 0.1% Triton-X-100 in PBS for 20 min. and then blocked with the appropriate serum for 30 min at room temperature. Plates were incubated overnight with primary antibodies (4 °C), rewarmed (37 °C) for 45 min, washed (3×) with PBS (5 min), and incubated with the corresponding fluorescent secondary antibody. The nuclei were labelled with 4′,6-diamidino- 2-phenylindole (DAPI), and images were collected with a Leica (SP8) confocal microscopy system at 400 × magnification.

### ELISA

A mouse Collagen I alpha 1 ELISA Kit (Cusabio, Wuhan, China) was used according to the manufacturer’s instructions to determine Collagen I levels. Plasma samples were collected to evaluate brain natriuretic peptide 45 (BNP 45) using a BNP 45 mouse ELISA Kit (RayBiotech, Georgia, USA).

### RNA isolation and reverse transcription-polymerase chain reaction (RT-PCR)

Total RNA from the border zone of left ventricular myocardial tissues and CFs was isolated using TRIzol reagent (Invitrogen, Carlsbad, CA, USA). One microgram of total RNA was reverse transcribed with a First-Strand cDNA Synthesis Kit (Takara), and the cDNA samples were amplified with PCR (KAPA SYBR^®^ rapid quantitative PCR kit, KK4600) using specific primers. The primer sequences and annealing temperatures used in this study are listed in Supplemental Table S2 (see Supplemental Table S2).

### Western blot analysis

Total tissue protein was obtained from the border zone of the left ventricular myocardial tissue; cellular protein was obtained from the treated cardiac fibroblasts. The protein concentration was determined with a BCA. Equal amounts of total protein were separated with 8–10% SDS-PAGE and blotted onto PVDF membranes (Millipore, Bedford, MA, USA). Membranes were blocked with 5% BSA, incubated overnight at 4 °C with the antibodies of interest, washed (10 min, 3×) with TBST, incubated with the corresponding secondary antibody (2 h, room temperature), and washed with TBST buffer (10 min, 3×); this was followed by detection with a Western Blotting Luminol Reagent (ECL) kit (Amersham, NJ, USA). Detection of phosphorylated proteins was performed first, and then PVDF membranes were stripped with stripping buffer (Beyotime Biotech, Haimen, Jiangsu, China) according to manufacturer’s instructions; the second hybridization was carried out to measure the levels of total protein. The protein bands were visualized (Protein Simple system, Santa Clara, California), and the intensity of the immunoblot bands was quantified with densitometry image analysis software (Quantity One, Bio-Rad, Hercules, California). Densitometry values were normalized to the GAPDH level in each sample.

### Statistical Analysis

The means ± SE (standard error) were calculated for all data variables. Group differences were evaluated using a one-way ANOVA test. All analyses were performed using SPSS (v19, SPSS Inc., Chicago, Illinois, USA), with p < 0.05 considered statistically significant.

## Additional Information

**How to cite this article:** Zhang, J. *et al*. Targeted inhibition of Focal Adhesion Kinase Attenuates Cardiac Fibrosis and Preserves Heart Function in Adverse Cardiac Remodeling. *Sci. Rep.* **7**, 43146; doi: 10.1038/srep43146 (2017).

**Publisher's note:** Springer Nature remains neutral with regard to jurisdictional claims in published maps and institutional affiliations.

## Supplementary Material

Supplementary Information

Supplementary Figures

## Figures and Tables

**Table 1:** Echocardiographic analysis and tissue harvest in Sham and CAL mice.

		Sham^a^	Sham + PF^b^	MI^c^	MI + PF^d^
BW		48.79 ± 0.69	49.26 ± 1.61	46.13 ± 0.70	47.59 ± 0.78
TL		10.77 ± 0.08	10.78 ± 0.12	10.41 ± 0.07	10.59 ± 0.11
HW		0.21 ± 0.01	0.23 ± 0.01	0.25 ± 0.01*	0.22 ± 0.01^#^
LW		0.23 ± 0.01	0.26 ± 0.02	0.37 ± 0.02*	0.32 ± 0.01^#^
HW/BW		4.37 ± 0.16	4.72 ± 0.19	5.48 ± 0.31*	4.66 ± 0.19^#^
LW/BW		4.71 ± 0.20	5.23 ± 0.28	8.04 ± 0.44*	6.69 ± 0.21^#^
BNP		120.06 ± 9.86	117.62 ± 15.53	515.17 ± 50.75*	399.03 ± 41.37^#^
	Day 30	68.48 ± 3.80	65.84 ± 2.34	18.89 ± 1.33*	27.87 ± 1.31^#^
LVEF	Day 60	70.48 ± 2.12	75.52 ± 2.08	15.26 ± 2.21*	22.22 ± 2.10^#^
	Day 90	68.93 ± 2.67	71.43 ± 4.66	12.42 ± 0.88*	22.30 ± 1.62^#^
	Day 30	38.26 ± 3.12	36.08 ± 1.78	8.73 ± 0.64*	13.21 ± 0.67^#^
FS	Day 60	39.48 ± 1.58	43.90 ± 1.71	7.03 ± 1.06*	10.39 ± 1.04^#^
	Day 90	38.44 ± 2.06	41.05 ± 3.71	5.67 ± 0.41*	10.58 ± 0.74^#^
	Day 30	22.23 ± 4.56	27.87 ± 2.57	146.01 ± 14.80*	117.51 ± 6.79^#^
LV Vol;s	Day 60	20.77 ± 3.78	17.07 ± 2.61	165.85 ± 13.13*	133.56 ± 11.99^#^
	Day 90	23.11 ± 2.82	21.83 ± 4.69	196.45 ± 13.05*	123.72 ± 10.43^#^

CAL: coronary artery ligation; MI: myocardial infarction; PF: treated with PF-573228; BW: body weight; TL: tail length; HW: heart weight; LW: lung weight; BNP: brain natriuretic peptide(pg/ml); LVEF: left ventricular ejection fraction; FS: fractional shortening; LV Vol; s: systolic left ventricular volume; a: n = 6; b: n = 6; c: n = 10 (1 month), n = 9(2 month), n = 8(3 month);d:n = 10 (1 month), n = 10 (2 month), n = 9 (3 month); Values are means ± SEM of three separate M-mode measurements. *p < 0.05 vs. Sham & Sham + PF ^#^P < 0.05 vs. MI.
