# Supplementary material for: Targeted inhibition of Focal Adhesion Kinase Attenuates Cardiac Fibrosis and Preserves Heart Function in Adverse Cardiac Remodeling
Source: Sci Rep. 2017 Feb 22;7:43146. doi: 10.1038/srep43146 (PMC5320468; doi:10.1038/srep43146)
Supplement: Supplementary Information [file srep43146-s1.pdf]

Targeted inhibition of Focal Adhesion Kinase Attenuates Cardiac Fibrosis and Preserves Heart Function in Adverse Cardiac Remodeling

Jie Zhang[1], Guangpu Fan[2], Hui Zhao[1], Zhiwei Wang[1], Fei Li[1], Peide Zhang[1], Jing Zhang[1], Xu Wang[1], Wei Wang[1*]

Supplementary Information

Supplementary table S1 the description and dilute concentration of the antibody used in this study

Supplementary table S2 Primer sequences and product sizes for predicted genes

Supplementary table S3 Echocardiographic analysis at the 7th day after MI

Supplementary table S4 Echocardiographic analysis at the first month after treatment

Supplementary table S5 Echocardiographic analysis at the second month after treatment

Supplementary table S6 Echocardiographic analysis at the third month after treatment

Supplementary table S2 the dilute concentration of the antibody used in this study

	Purchased from	Description	Dilution
**Collagen I**	Abcam(34710)	Rabbit/ polyclonal	WB 1:1000<br>IHC 1:1500<br>IF 1:200
**Collagen III**	Abcam(7778)	Rabbit/ polyclonal	IHC 1:500<br>IF 1:200
**Fibronectin**	Abcam(2413)	Rabbit/ polyclonal	WB 1:1000<br>IHC 1:500<br>IF 1:150
**Elastin**	Abcam(21610)	Rabbit/ polyclonal	IHC 1:500<br>IF 1:200
**p-FAK**	Abcam(81298)	Rabbit/ monoclonal	IF 1:200
**p-FAK**	CST(3283)	Rabbit/ polyclonal	WB 1:1000
**FAK**	CST(3285)	Rabbit/ polyclonal	IHC 1:100<br>WB 1:1000
**Vimentin**	Abcam(8978)	Mouse/ monoclonal	IF: 1:200
**Vimentin**	Abcam(92547)	Rabbit/ polyclonal	WB 1:1000
**α -SMA**	Abcam(5694)	Rabbit/ polyclonal	WB 1:1000
**α -SMA**	Abcam(7817)	Mouse/ monoclonal	WB 1:200
**LOX**	Abcam(31238)	Rabbit/ polyclonal	WB 1:500
**t-P70/S6K**	CST (9234s)	Rabbit/ polyclonal	WB 1:1000
**p-P70/S6K**	CST (2708s)	Rabbit/ polyclonal	WB 1:1000
**p-AKT**	CST (4060s)	Rabbit/ polyclonal	WB 1:1000
**t-AKT**	CST (4691s)	Rabbit/ polyclonal	WB 1:1000
**p-mTOR**	CST (2974p)	Rabbit/ polyclonal	WB 1:1000
**t-mTOR**	CST (2983p)	Rabbit/ polyclonal	WB 1:1000
**p-ERK1/2**	CST (4377)	Rabbit/ polyclonal	WB 1:1000
**t-ERK1/2**	CST (4695)	Rabbit/ polyclonal	WB 1:1000
**GAPDH**	ZSGB-BIO(TA336621)	mouse/ monoclonal	WB 1:1000
**Anti-Rabbit IgG H&L (Alexa Fluor® 488)**	Abcam (150077)	Goat/ Polyclonal	IF 1:400
**Alexa Fluor® 488 AffiniPure Goat Anti-Mouse IgG (H+L)**	Jackson(115545003)	Goat/ Polyclonal	IF 1:400
**Alexa Fluor® 594 AffiniPure Goat Anti-Rabbit IgG (H+L)**	Jackson(111585003)	Goat/ Polyclonal	IF 1:400

Supplementary table S2 : Primer sequences and product sizes for predicted genes

Gene	Primer	Sequence (5' to 3')	Length
**FAK**	Forward	GGCTGTCATCGAGATGTCCA	114bp
	Reverse	TCTCATCCACCGTGGCTAGT	
**Vimentin**	Forward	ATTTCTCTGCCTCTGCCAAC	113bp
	Reverse	CAACCGTCTTAATCAGGAGTGT	
**α-SMA**	Forward	TACCACCATGTACCCAGGCA	152bp
	Reverse	GAAGGTAGACAGCGAAGCCA	
**cta**	Forward	GACGAGATGGCATCCCTG	141bp
	Reverse	CACGGAAACTCCAGCTGATT	
**LOX**	Forward	CCACAGCATGGACGAATTCA	191bp
	Reverse	GTCTGCCGCATAGGTGTCAT	
**Fibronectin**	Forward	ACATGTCAGACCTGCCTTGG	121bp
	Reverse	GGTGAGATTGAAGTGGGAGC	
**Laminin**	Forward	TGTGAGCTGTGTGCTTCTGG	139bp
	Reverse	GCACCGGAAATCACTGTCAC	
**Elastin**	Forward	CTAAAGCAGCCGCCAAAG	150bp
	Reverse	AGGACCAGCTCCAATACCAG	
**β-actin**	Forward	ACTCATCGTACTCCTGCTTGCT	130bp
	Reverse	GCTAGGCATTTCCATCTTCA	

Supplementary table S3 Echocardiographic analysis at the 7th day after MI

	Sham	Sham +PF	MI	MI+PF
**LVEF**	68.82±2.15	67.73±2.30	48.05±1.74*	43.96±1.40#
**FS**	38.25±1.66	37.64±1.73	24.26±1.06*	21.99±0.81#
**LV mass**	152.93±9.08	175.49±10.27	143.04±10.12	167.44±14.64
**LV mass (correct)**	122.34±7.26	140.39±8.21	114.43±8.09	133.95±11.72
**LV Vol; d**	73.72 ±7.02	88.34 ±5.67	105.72 ±3.41*	138.64 ±6.70#
**LV Vol; s**	23.34±3.57	28.92±3.54	55.10±3.06*	78.21±5.17#
**LVAW,s**	1.61±0.05	1.71±0.06	0.87±0.11*	0.67±0.13
**LVAW, d**	1.01±0.04	1.07±0.04	0.67±0.07*	0.51±0.09
**LVID, d**	4.07±0.16	4.40±0.12	4.76±0.06*	5.34±0.11#
**LVID,s**	2.52±0.14	2.75±0.14	3.61±0.08*	4.17±0.12#
**LVPW, d.**	0.89±0.03	0.84±0.04	0.81±0.03	0.92±0.06
**LVPW,s**	1.38±0.07	1.28±0.08	1.25±0.04	1.46±0.07#

Table 3. Echocardiographic analysis at the 7th day after MI model were established, Values are means ±SEM of three separate M-mode measurements. LVEF: left ventricular ejection fraction; FS: fractional shortening; LV mass : left ventricular mass; LV Vol; s: systolic left ventricular volume; LV Vol;d :diastolic left ventricular volume; LVAW,s : systolic left ventricular anterior wall; LVAW,d: diastolic left ventricular posterior wall; LVID,d: diastolic left ventricular internal dimension; LVID,s : systolic left ventricular internal dimension; LVPW,d: diastolic left ventricular posterior wall; LVPW,s: systolic left ventricular posterior wall.* $p<0.05$ vs. Sham＆Sham+PF; #$P< 0.05$ vs. MI.

Supplementary table S4 Echocardiographic analysis at the first month after treatment

	Sham	Sham +PF	MI	MI+PF
**LVEF**	68.48±3.80	65.84±2.34	18.89±1.33*	27.87±1.31#
**FS**	38.26±3.12	36.08±1.78	8.73±0.64*	13.21±0.67#
**LV mass**	160.07±12.60	166.37±7.03	155.18±20.37	193.88±19.59
**LV mass (correct)**	128.06±10.08	133.10±5.63	126.31±15.88	155.10±15.67
**LV Vol; d**	67.70±7.31	81.62±5.67	179.54±17.29*	162.46±7.88
**LV Vol; s**	22.23±4.56	27.87±2.57	146.01±14.80*	117.51±6.79#
**LVAW,s**	1.72±0.11	1.64±0.06	0.49±0.04*	0.45±0.07#
**LVAW, d**	1.16±0.07	1.06±0.06	0.41±0.03*	0.40±0.07
**LVID, d**	3.92±0.18	4.26±0.13	5.94±0.25*	5.72±0.12
**LVID,s**	2.43±0.21	2.72±0.11	5.42±0.24*	4.97±0.13
**LVPW, d.**	0.88±0.07	0.86±0.04	0.70±0.06*	1.07±0.10#
**LVPW,s**	1.33±0.10	1.39±0.05	1.00±0.10*	1.40±0.10#

Table 1. Echocardiographic analysis at the first month after implantation of the ALZET mini-osmotic pump, Values are means ± SEM of three separate M-mode measurements. LVEF: left ventricular ejection fraction; FS: fractional shortening; LV mass : left ventricular mass; LV Vol; s: systolic left ventricular volume; LV Vol;d :diastolic left ventricular volume; LVAW,s : systolic left ventricular anterior wall; LVAW,d: diastolic left ventricular posterior wall; LVID,d: diastolic left ventricular internal dimension; LVID,s : systolic left ventricular internal dimension;LVPW,d: diastolic left ventricular posterior wall; LVPW,s: systolic left ventricular posterior wall.* $p<0.05$ vs. Sham＆Sham+PF; #$P< 0.05$ vs. MI.

`

Supplementary table S5 Echocardiographic analysis at the second month after treatment

	Sham	Sham +PF	MI	MI+PF
**LVEF**	70.48±2.12	75.52±2.08	15.26±2.21*	22.22±2.10#
**FS**	39.48±1.58	43.90±71	7.03±1.06*	10.39±1.04#
**LV mass**	161.11±19.19	174.51±12.26	170.62±17.86	144.38±19.24
**LV mass (correct)**	128.89±15.35	139.61±9.81	136.49±14.29	115.50±15.39
**LV Vol; d**	68.19±7.46	68.32±68.32	194.65±12.14*	171.30±14.64
**LV Vol; s**	20.77±3.78	17.07±2.61	165.85±13.13*	133.56±11.99#
**LVAW,s**	1.55±0.06	1.91±0.06	0.37±0.04*	0.39±0.04
**LVAW, d**	1.02±0.05	1.19±0.05	0.34±0.05*	0.36±0.03
**LVID, d**	3.93±0.18	3.65±0.41	6.19±0.16*	5.83±0.22
**LVID,s**	2.39±0.17	2.22±0.13	5.76±0.19*	5.22±0.21#
**LVPW, d.**	1.01±0.07	0.96±0.04	0.84±0.10	0.73±0.08
**LVPW,s**	1.56±0.09	1.53±0.03	1.06±0.12*	0.98±0.12

Table 1. Echocardiographic analysis at the second month after implantation of the ALZET mini-osmotic pump, Values are means ±SEM of three separate M-mode measurements. LVEF: left ventricular ejection fraction; FS: fractional shortening; LV mass : left ventricular mass; LV Vol; s: systolic left ventricular volume; LV Vol;d :diastolic left ventricular volume; LVAW,s : systolic left ventricular anterior wall; LVAW,d: diastolic left ventricular posterior wall; LVID,d: diastolic left ventricular internal dimension; LVID,s : systolic left ventricular internal dimension;LVPW,d: diastolic left ventricular posterior wall; LVPW,s: systolic left ventricular posterior wall.* $p<0.05$ vs. Sham&Sham+PF; #$P<0.05$ vs. MI.

Supplementary table S6 Echocardiographic analysis at the third month after treatment

	Sham	Sham +PF	MI	MI+PF
**LVEF**	68.93±2.67	71.43±4.66	12.42±0.88*	22.30±1.62#
**FS**	38.44±2.06	41.05±3.71	5.67±0.41*	10.58±0.74#
**LV mass**	159.48±3.72	160.88±18.07	154.04±23.37	155.95±11.11
**LV mass (correct)**	127.59±10.97	128.70±14.46	123.23±18.70	124.76±8.89
**LV Vol; d**	73.90±5.73	73.17±6.45	223.66±13.28*	158.73±0.62#
**LV Vol; s**	23.11±2.82	21.83±4.69	196.45±13.05*	123.72±10.43#
**LVAW,s**	1.56±0.11	1.70±0.09	0.29±0.05*	0.42±0.07
**LVAW, d**	1.03±0.05	1.09±0.07	0.27±0.04*	0.38±0.05
**LVID, d**	4.08±0.13	4.06±0.15	6.58±0.18*	5.66±0.16#
**LVID,s**	2.51±0.12	2.41±0.22	6.21±0.19*	5.07±0.18#
**LVPW, d.**	0.91±0.04	0.86±0.06	0.70±0.11	0.87±0.07
**LVPW,s**	1.45±0.06	1.39±0.08	0.82±0.12*	1.12±0.09#

Table 1. Echocardiographic analysis at the third month after implantation of the ALZET mini-osmotic pump, Values are means ±SEM of three separate M-mode measurements. LVEF: left ventricular ejection fraction; FS: fractional shortening; LV mass : left ventricular mass; LV Vol; s: systolic left ventricular volume; LV Vol;d :diastolic left ventricular volume; LVAW,s : systolic left ventricular anterior wall; LVAW,d: diastolic left ventricular posterior wall; LVID,d: diastolic left ventricular internal dimension; LVID,s : systolic left ventricular internal dimension;LVPW,d: diastolic left ventricular posterior wall; LVPW,s: systolic left ventricular posterior wall.* p<0.05 vs. Sham&Sham+PF; #P< 0.05 vs. MI.
