# Supplementary material for: Targeted inhibition of Focal Adhesion Kinase Attenuates Cardiac Fibrosis and Preserves Heart Function in Adverse Cardiac Remodeling
Source: Sci Rep. 2017 Feb 22;7:43146. doi: 10.1038/srep43146 (PMC5320468; doi:10.1038/srep43146)

Targeted inhibition of Focal Adhesion Kinase Attenuates Cardiac Fibrosis and Preserves Heart Function in Adverse Cardiac Remodeling

Jie Zhang[1], Guangpu Fan[2], Hui Zhao[1], Zhiwei Wang[1], Fei Li[1], Peide Zhang[1], Jing Zhang[1], Xu Wang[1], Wei Wang[1*]

Supplementary Information

Supplementary Figure S1   Original blots used in the figure 1(1a, 1b)

Supplementary Figure S2   Original blots used in the figure 2a

Supplementary Figure S3   Original blots used in the figure 3a

Supplementary Figure S5   Original blots used in the figure 5a

Supplementary Figure S6   Multiple exposures of original blots used in the main figures

Supplementary Figure S1a

p-FAK 125KDa

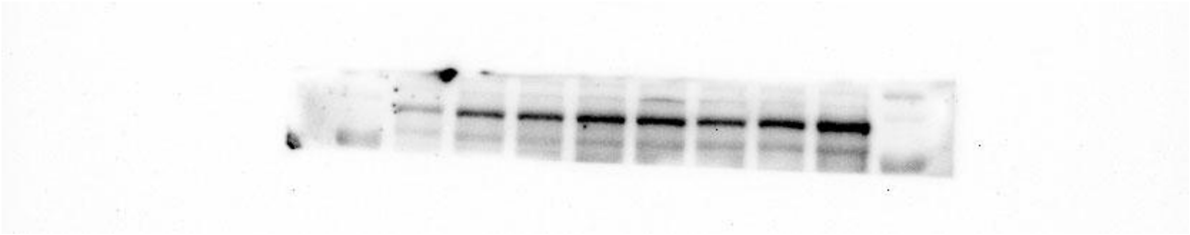

t-FAK 125KDa

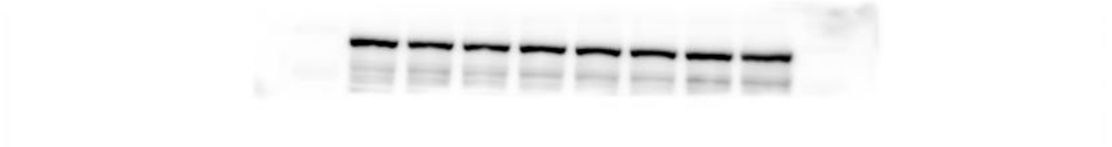

α-SMA 42KDa

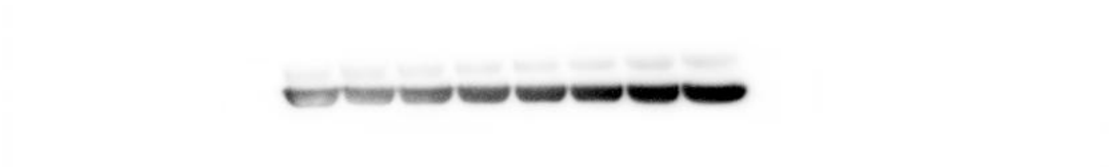

Vimentin 54KDa

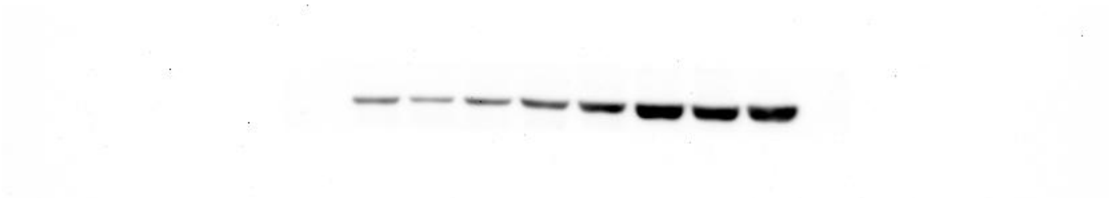

GAPDH 37KDa

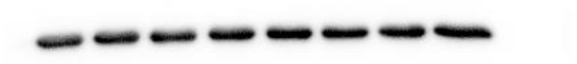

Supplementary Figure S 1b

P-FAK (PF-573,228 concentration) 125KDa

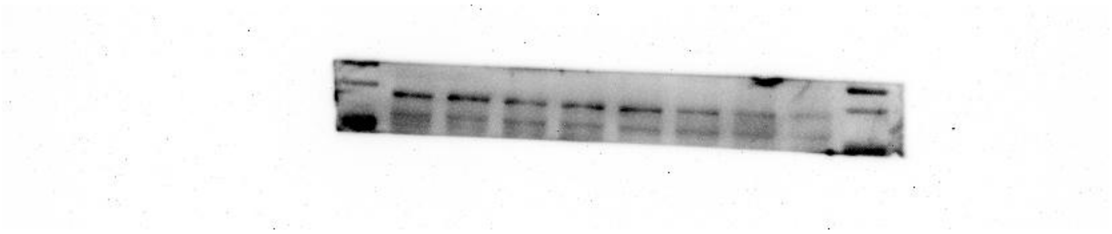

t-FAK (PF-573,228 concentration) 125KDa

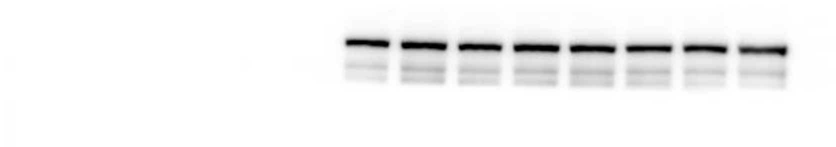

GAPDH(PF-573,228 concentration) 37KDa

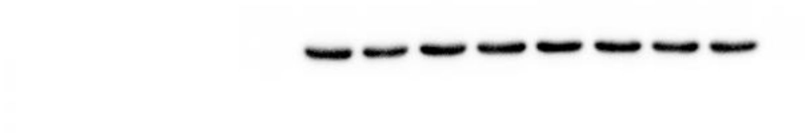

Supplementary Figure S2

p-FAK 125KDa

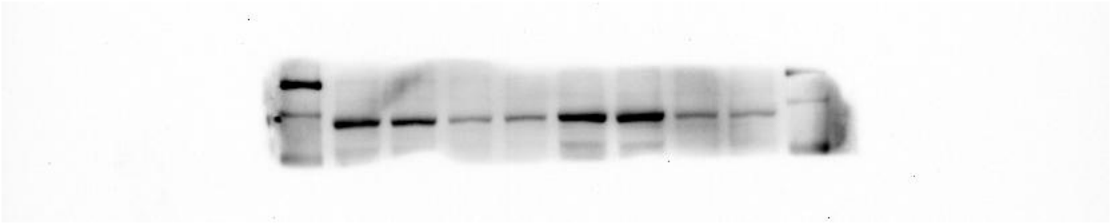

t-FAK 125KDa

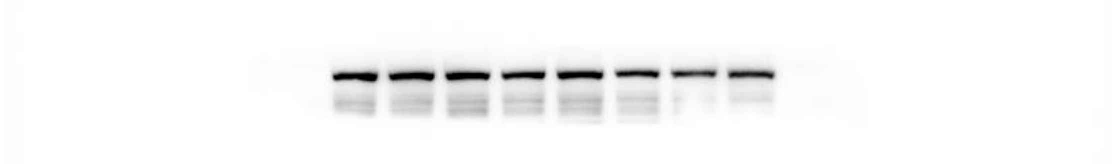

Vimentin 54KDa

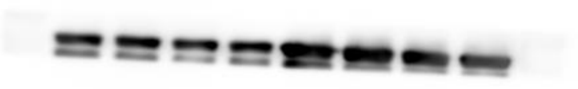

α-SMA 42KDa

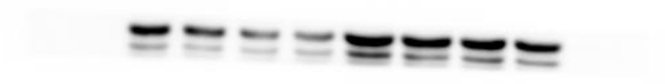

GAPDH   37KDa

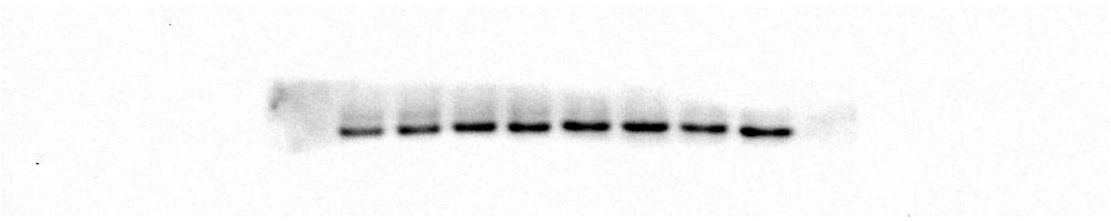

Supplementary Figure S3

Collagen-1   130KDa

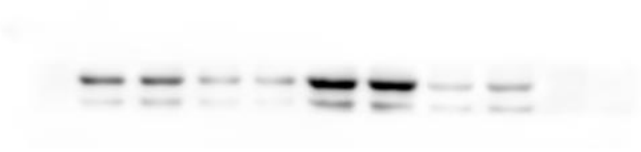

Fibronectin   262 KDa

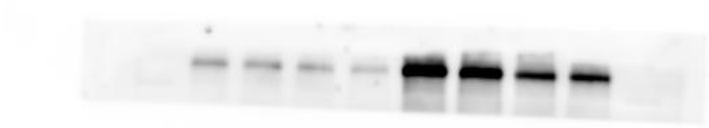

Laminin   250KDa

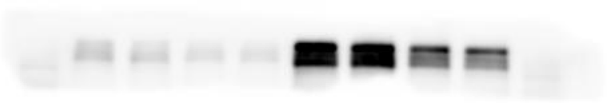

LOX   262 KDa

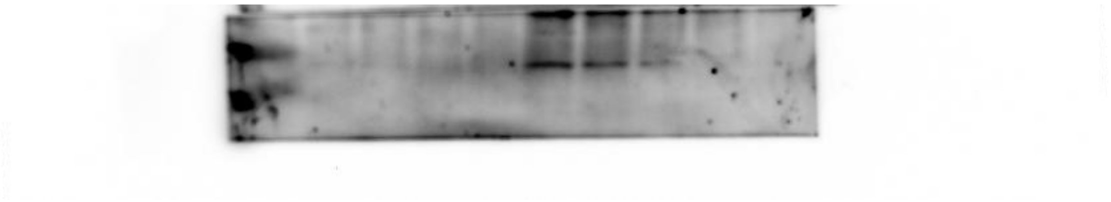

GAPDH   37 KDa

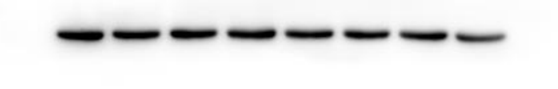

Supplementary Figure S5a

p-P70s6k 70, 85 KDa

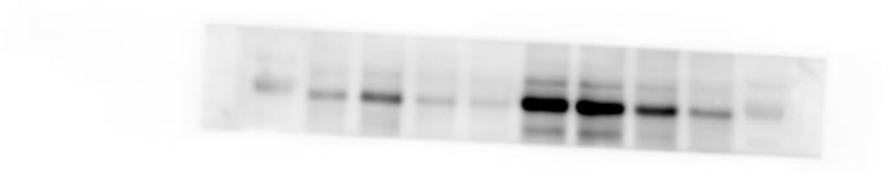

t-P70S6K 70, 85 KDa

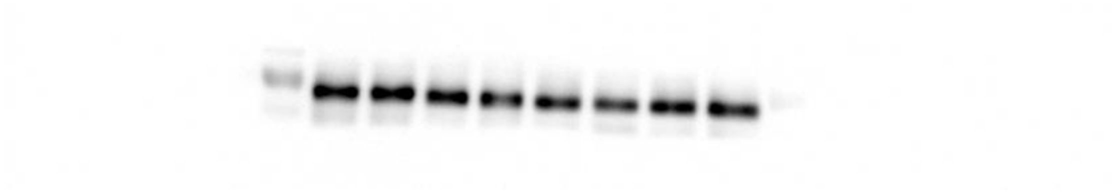

p-Akt 60 KDa

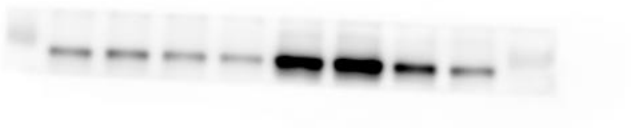

T-AKT 60 KDa

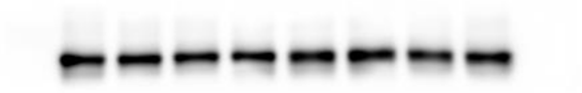

p-mTOR 289 KDa

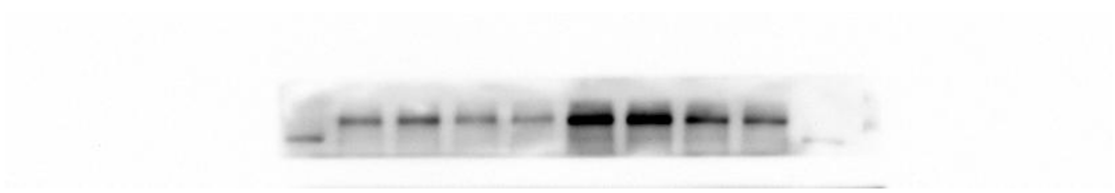

t-mTOR 289KDa

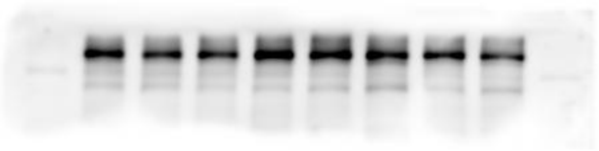

p-ERK 42, 44 KDa

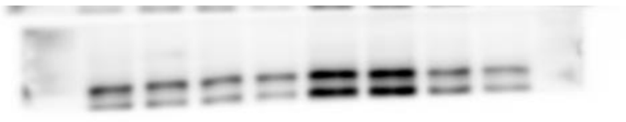

t-ERK 42, 44 KDa

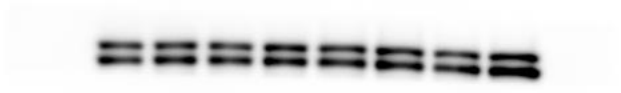

GAPDH 37 KDa

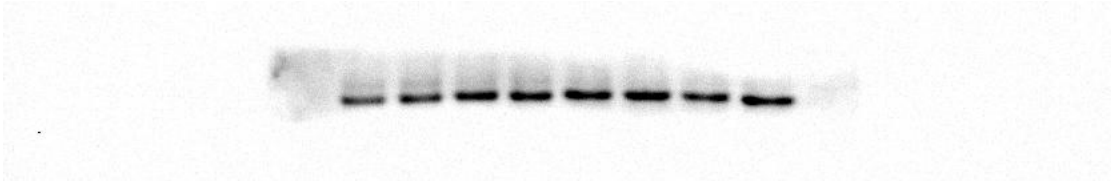

Supplementary Figure S6

LOX 262 KDa Exposure time: 1min

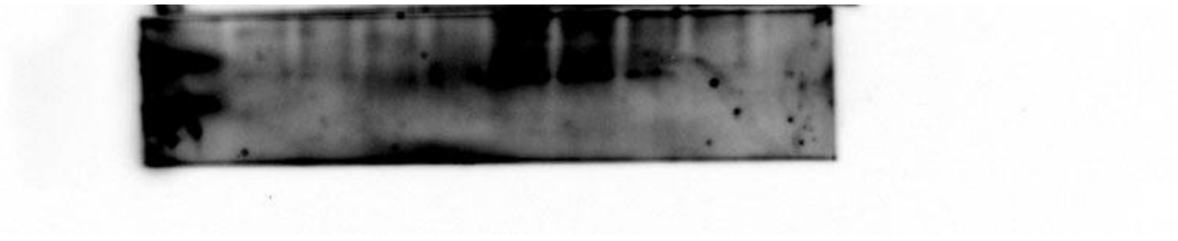

LOX 262 KDa Exposure time: 2min14s

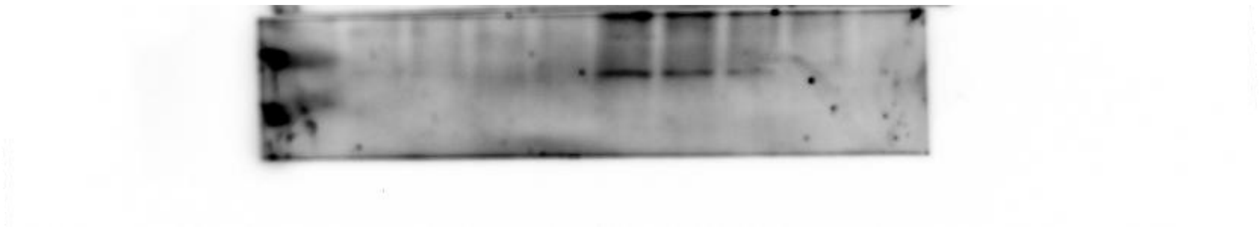

Vimentin 54KDa Exposure time: 54s

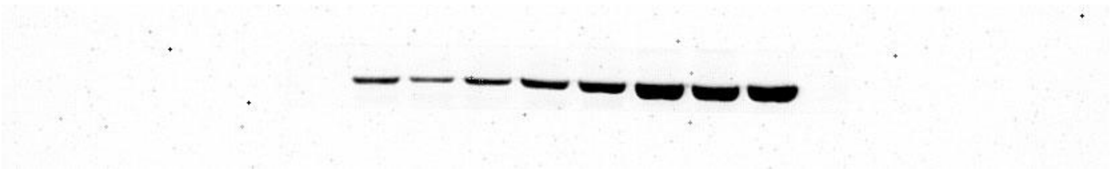

Vimentin 54KDa Exposure time: 1min36s

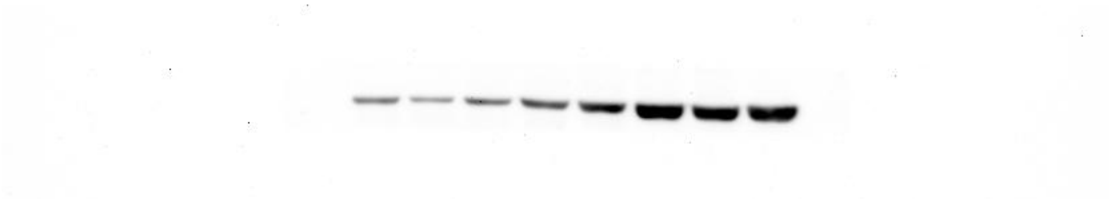

GAPDH 37KDa Exposure time: 54s

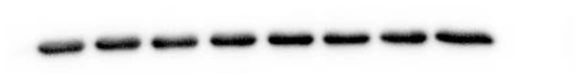

GAPDH 37KDa Exposure time: 1min36s

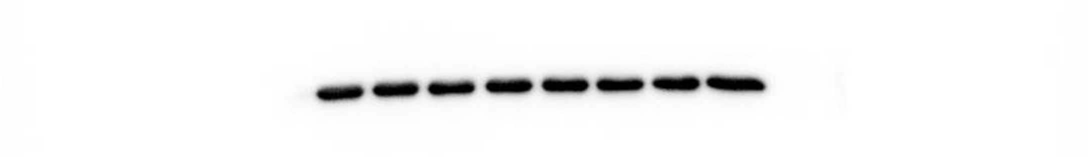

t-ERK 42, 44 KDa Exposure time: 54s

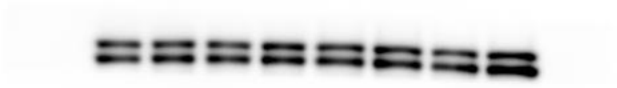

t-ERK 42, 44 KDa Exposure time: 1min36s

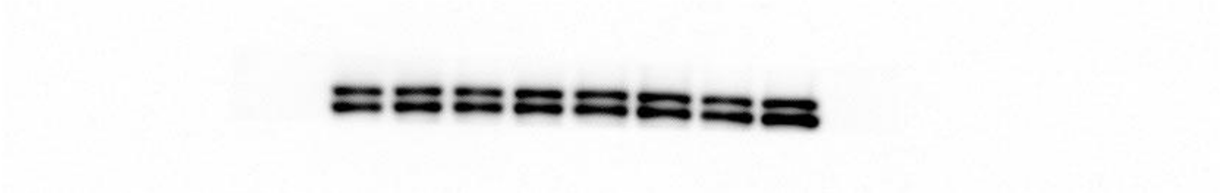

Supplement: Supplementary Figures [file srep43146-s2.pdf]
